# Nosological profile of dermatological diseases in primary health care and dermatology secondary care in Florianópolis (2016–2017)^☆^^☆☆^

**DOI:** 10.1016/j.abd.2020.01.004

**Published:** 2020-05-16

**Authors:** Iago Gonçalves Ferreira, Dannielle Fernandes Godoi, Elaine Regina Perugini

**Affiliations:** a Department of Dermatology, Universidade Federal de Ciências da Saúde de Porto Alegre, Porto Alegre, RS, Brazil; b Department of Clinical Medicine, Universidade Federal de Santa Catarina, Florianópolis, SC, Brazil; c Department of Dermatology, Faculdade de Medicina de Marília, Marília, SP, Brazil; d Service of Dermatology, Secretaria Municipal de Saúde de Florianópolis, Florianópolis, SC, Brazil

**Keywords:** Dermatology, Diagnosis, epidemiology, Epidemiology, descriptive, Physicians, primary care, Primary health care

## Abstract

**Background:**

Dermatology encompasses the management of many disorders of the skin and cutaneous appendages, making the analysis of epidemiological profiles relevant for health planning.

**Objective:**

The study aims to describe the nosological profile of dermatological diseases in Florianopolis, analyzing the interrelation among the primary health care and dermatology services, from January 2016 to December 2017.

**Method:**

Descriptive study from records of medical visits from the primary health care and dermatology services, as well as records of reports issued by the teledermatology service.

**Results:**

In primary health care, from 55,265 medical visits – 28,546 in 2016 and 26,719 in 2017, there was a higher prevalence of “Atopic dermatitis” (6.38%), “other disorders of skin and subcutaneous tissue” (5.10%), and “Scabies” (4.55%). In dermatology secondary care, from 19,964 visits – 10,068 in 2016 and 9626 in 2017, the most prevalent diagnoses were “Other malignant neoplasms of the skin” (14.75%) and “Skin changes due to chronic exposure to nonionizing radiation” (10.20%).

**Study limitations:**

Some dermatological consultations in primary health care may have been under-registered due to the attribution of non-specific or overly broad diagnoses.

**Conclusion:**

This study presents different nosological profiles of skin diseases in primary health care and dermatology secondary care, reinforcing the importance of primary health care's role in the management of less complex conditions, referring more complex cases that require more specialized experience to dermatology services..

## Introduction

Skin is an extensive human organ responsible for the interface between organisms and the environment, constituting an important element in human interrelationships. Dermatological diseases are among the leading causes of global burden of diseases, having major repercussions on the quality of life of millions of people worldwide.^1^, ^2^, ^3^

Despite their epidemiological and social impact, dermatological diseases are still neglected by health policy makers. This negligence occurs due to both underestimation of their morbidity and mortality and issues inherent to medical education, which in many cases lacks suitable instruction and practice in dermatology.^1^, ^4^, ^5^

Dermatology is the medical specialty responsible for managing disorders of the skin, mucous membranes, and cutaneous appendages. Dermatological examination can identify changes in skin colour and texture, as well as lesions with different distributions and morphologies.^2^, ^6^, ^7^, ^8^ Dermatologists show higher diagnostic accuracy in the evaluation of potentially malignant and rarer diseases, with a tendency towards broader differential diagnoses than non-dermatologists.^4^

In light of this situation, general practitioners (GPs) must demonstrate suitable skills to diagnose and manage the most prevalent skin diseases in primary health care (PHC), which is the gateway to other health care levels.^5^, ^9^ Despite having the required skills, there is a tendency among GPs to minimize or confuse some skin diseases, overdiagnosing more common conditions such as eczema, warts, and infectious diseases, to the detriment of more complex diagnoses, including cancer. Thus, they may contribute to delays in treatment initiation and consequently, worse prognoses.^5^

Brazil has different sociodemographic realities, partly due to its continental dimensions, which directly impact the epidemiologic profile of dermatological diseases. The diverse context and difficulties in managing of these conditions in PHC represent a major challenge for dermatological care within health systems. In doing so, it is necessary to develop studies which evaluate the spectrum of the Brazilian epidemiological situation, contributing to planning and health resource allocation.^2^, ^5^, ^10^, ^11^, ^12^

Thus, facing the lack of studies about the epidemiological profiles of skin diseases, including those seen in PHC, this study aimed to describe the nosological profile of dermatological assistance in Florianopolis, in PHC and dermatology practices, from January 2016 to December 2017. This study aimed to understand the nosological scenario at these levels of attention, which will influence the knowledge and skills needed by general practitioners and dermatologists.

## Methods

### Scenario and context: Florianopolis municipal health services

Florianopolis, capital of the state of Santa Catarina, has an estimated population of 492,977 inhabitants, with a Human Development Index (HDI) of 0.847.^13^ The PHC consists of 49 health centres – distributed in four districts: Central, North, Inland, and South – organized under the Family Health Strategy (FHS) model, with coverage ranging from 88% to 100% during the period evaluated by this study.^14^

Dermatology service is distributed among three of four municipal polyclinics; it is responsible for outpatient care and teledermatology service reports. Telediagnostic dermatology was implemented in the city in 2015, promoting the organization of the referral flow from health centres, based on risk stratification criteria and colour classifications: white, blue, green, yellow, and red.^15^ The categories of this classification evolve progressively according to severity, starting with the white and blue categories, managed in PHC without need for referral; green and yellow, which require normal waiting time or urgent waiting time, respectively; and red, which needs immediate referral to secondary services.^16^

### Ethical aspects

The study was conducted based on principles of the Declaration of Helsinki and the Nuremberg Code, in line with the Research Standards Involving Human Beings of the National Research Ethics Commission (Res. CONEP 466/2012). Data collection had begun after approval from Research Ethics Committee of the Santa Catarina State Health Department (CAAE: 91700318.3.0000.0115), approval from the Committee of Health Research Project Monitoring of the Health Department of Florianopolis, and from the Santa Catarina Telemedicine Office, the institutions that provided the research data.

### Study design, data collection, and analysis

The authors conducted an analytical, observational, cross-sectional study, using secondary data from medical consultations records in PHC, teledermatology, and dermatology secondary care. Data were extracted from the systems: “*Infosaúde*” – from the Health Department of Florianopolis – composed of consultation records from PHC and dermatology services; and the Santa Catarina State Integrated Telemedicine and Telehealth System – from the Santa Catarina State Health Department – which stores teledermatology reports.

Data collection was conducted from August to October 2018, through records of dermatological diseases treated by doctors, in PHC and at dermatology service, based on the International Classification of Diseases (ICD). In order to allow an adequate understanding about interaction among health care levels, data were collected about risk classifications attributed in the teledermatology reports – the platform that regulates access to dermatology.

From the total of diagnoses identified through ICD version 10, the inclusion criteria were ICD categories which included diseases with mainly or predominantly dermatological involvement, organized into 12 classes according to table 1, adopting as a reference the previous epidemiological studies conducted by Brazilian Society of Dermatology (BSD) – in 2006^17^ and 2018^2^ – as well as the Dermatology in Primary Health Care Manual, produced by the Ministry of Health in 2002.^18^ The present study excluded the ICD categories of sexually transmitted diseases which have skin involvement exclusively in the urogenital and anal regions, considering that they would not represent a predominant dermatology field. Through these criteria, 121 disease categories were selected (5.91%) from the 2045 categories that compose the ICD version 10.

**Table 1:** Categories from the International Statistical Classification of Diseases (ICD) selected for research of dermatological treatment in the primary health care (PHC) and dermatology services of Florianopolis, in 2016 and 2017.^a^

Classifications	Categories
Skin and subcutaneous tissue infections (L00–L08)	(L00) Staphylococcal scalded skin syndrome
	(L01) Impetigo
	(L02) Cutaneous abscess, furuncle and carbuncle
	(L03) Cellulitis
	(L04) Acute lymphadenitis
	(L05) Pilonidal cyst
	(L08) Other local infections of skin and subcutaneous tissue
Bullous disorders (L10–L14)	(L10) Pemphigus
	(L11) Other acantholytic disorders
	(L12) Pemphigoid
	(L13) Other bullous disorders
	(L14) Bullous disorders in diseases classified elsewhere
Dermatitis and eczema (L20–L30)	(L20) Atopic dermatitis
	(L21) Seborrheic dermatitis
	(L22) Diaper [napkin] dermatitis
	(L23) Allergic contact dermatitis
	(L24) Irritant contact dermatitis
	(L25) Unspecified contact dermatitis
	(L26) Exfoliative dermatitis
	(L27) Dermatitis due to substances taken internally
	(L28) Lichen simplex chronicus and prurigo
	(L29) Pruritus
	(L30) Other dermatitis
Papulosquamous disorders (L40–L45)	(L40) Psoriasis
	(L41) Parapsoriasis
	(L42) Pityriasis rosea
	(L43) Lichen planus
	(L44) Other papulosquamous disorders
	(L45) Papulosquamous disorders in diseases classified elsewhere
Urticaria and erythema (L50–L54)	(L50) Urticaria
	(L51) Erythema multiforme
	(L52) Erythema nodosum
	(L53) Other erythematous conditions
	(L54) Erythema in diseases classified elsewhere
Radiation-related disorders of the skin and subcutaneous tissue (L55–L59)	(L55) Sunburn
	(L56) Other acute skin changes due to ultraviolet radiation
	(L57) Skin changes due to chronic exposure to nonionizing radiation
	(L58) Radiodermatitis
	(L59) Other disorders of skin and subcutaneous tissue related to radiation
Disorders of skin appendages (L60–L75)	(L60) Nail disorders
	(L62) Nail disorders in diseases classified elsewhere
	(L63) Alopecia areata
	(L64) Androgenic alopecia
	(L65) Other nonscarring hair loss
	(L66) Cicatricial alopecia (scarring hair loss)
	(L67) Hair colour and hair shaft abnormalities
	(L68) Hypertrichosis
	(L70) Acne
	(L71) Rosacea
	(L72) Follicular cysts of skin and subcutaneous tissue
	(L73) Other follicular disorders
	(L74) Eccrine sweat disorders
	(L75) Apocrine sweat disorders
Other skin and subcutaneous tissue disorders (L80–L99)	(L80) Vitiligo
	(L81) Other disorders of pigmentation
	(L82) Seborrheic keratosis
	(L83) Acanthosis nigricans
	(L84) Corns and callosities
	(L85) Other epidermal thickening
	(L86) Keratoderma in diseases classified elsewhere
	(L87) Transepidermal elimination disorders
	(L88) Pyoderma gangrenosum
	(L89) Decubitus ulcer and pressure area
	(L90) Atrophic disorders of skin
	(L91) Hypertrophic disorders of skin
	(L92) Granulomatous disorders of skin and subcutaneous tissue
	(L93) Lupus erythematosus
	(L94) Other localized connective tissue disorders
	(L95) Vasculitis limited to skin, not elsewhere classified
	(L97) Ulcer of lower limb, not elsewhere classified
	(L98) Other disorders of skin and subcutaneous tissue, not elsewhere classified
	(L99) aOther disorders of skin and subcutaneous tissue in diseases classified elsewhere
Neoplastic skin diseases^b^	(C43) Malignant melanoma of skin
	(C44) Other malignant neoplasms of skin
	(C46) Kaposi sarcoma
	(C84) Mature T/NK-cell lymphomas
	(D03) Melanoma in situ
	(D04) Carcinoma in situ of skin
	(D17) Benign lipomatous neoplasm
	(D18) Hemangioma and lymphangioma, any site
	(D22) Melanocytic naevi
	(D23) Other benign neoplasms of skin
	(D485) Neoplasm of uncertain or unknown behaviour of skin.
Leprosy^b^	(A30) Leprosy (Hansen disease)
	(B92) Sequelae of leprosy
Other skin disorders^b^	(E70.3) Albinism
	(I78.0) Hereditary haemorrhagic telangiectasia
	(I78.1) Naevus, non-neoplastic
	(Q82) Other congenital malformations of skin
Other skin infections^b^	(A18.4) Tuberculosis of skin and subcutaneous tissue
	(A22) Anthrax
	(A26) Erysipeloid
	(A31.1) Infection due to other mycobacteria
	(A32.0) Cutaneous listeriosis
	(A44.1) Cutaneous and mucocutaneous bartonellosis
	(A46) Erysipelas
	(A51.3) Secondary syphilis of skin and mucous membranes
	(A66) Multiple papillomata and wet crab yaws
	(A67) Pinta [carate]
	(A69.2) Lyme disease
	(B00.0) Eczema herpeticum
	(B00.1) Herpesviral vesicular dermatitis
	(B01) Varicella [chickenpox]
	(B02) Zoster [herpes zoster]
	(B07) Viral warts
	(B08) Other viral infections characterized by skin and mucous membrane lesions, not elsewhere classified
	(B09) Unspecified viral infection characterized by skin and mucous membrane lesions (B35) Dermatophytoses
	(B36.0) Pityriasis versicolor
	(B36.8/B36.9) Other specified superficial mycoses/Superficial mycosis, unspecified
	(B40.3) Cutaneous blastomycosis
	(B43.2) Subcutaneous phaeomycotic abscess and cyst
	(B49) Unspecified mycosis
	(B55.1) Cutaneous leishmaniasis
	(B55.2) Mucocutaneous leishmaniasis
	(B85) Pediculosis and phthiriasis
	(B86) Scabies
	(B87.0) Cutaneous myiasis
	(B88.1) Tungiasis [sandflea infestation]

a Elaborated by the authors based on previous studies.^2^, ^17^, ^18^

b No-L, ICD classifications.

### Data analysis

Data were analyzed using the descriptive method, with categorical and quantitative variables represented by graphs and tables developed in Microsoft Excel® 2010 and Microsoft Word® 2010. The categorical variables analysis “ICD category groups” between the health care consultation levels – PHC and dermatology – were performed using IBM SPSS v. 20, adopting the chi-squared test for comparative analysis between groups.

## Results

In 2016 and 2017, 1,029,961 records were collected from primary health care, 527,825 in 2016 and 502,136 in 2017. From these records, 55,265 records were identified with the above selected ICDs categories - 28,546 in 2016 and 26,719 in 2017, representing approximately 5.36% of PHC consultations. During this period, the dermatology service registered 23,478 medical visits – 12,130 in 2016 and 11,348 in 2017; of these records, 19,964 consultations had the selected ICD categories – 10,068 in 2016 and 9626 in 2017. Regarding referrals to the teledermatology service, a tool for organizing the flow between PHC and dermatology secondary care, the authors identified 3372 records in 2016 and 2921 in 2017.

Regarding ICD profiles, primary health care had the highest prevalence of ICDs such as “Atopic dermatitis” (L20), “Other skin and subcutaneous tissue disorders” (L98), and “Scabies” (B86) (Table 2).

**Table 2:** Main categories from the International Statistical Classification of Diseases – 10th Revision (ICD-10) treated in the primary health care service of Florianópolis, in 2016 and 2017.

Rank	ICD-10		*n*	%
1	L20	Atopic dermatitis	3531	6.38
2	L98	Other disorders of skin and subcutaneous tissue	2819	5.10
3	B86	Scabies	2518	4.55
4	L01	Impetigo	2371	4.29
5	L60	Nail disorders	2277	4.12
6	L30	Other dermatitis	2078	3.76
7	L29	Pruritus	1854	3.35
8	B49	Unspecified mycosis	1507	2.72
9	L72	Follicular cysts of skin and subcutaneous tissue	1495	2.70
10	L40	Psoriasis	1382	2.50
11	L23	Allergic contact dermatitis	1372	2.48
12	L50	Urticaria	1357	2.45
13	B07	Viral warts	1356	2.45
14	B36	Pityriasis versicolor	1072	1.94
15	B369	Other specified superficial mycoses	916	1.65
Total			55,265	100.00

**Figure 1:**
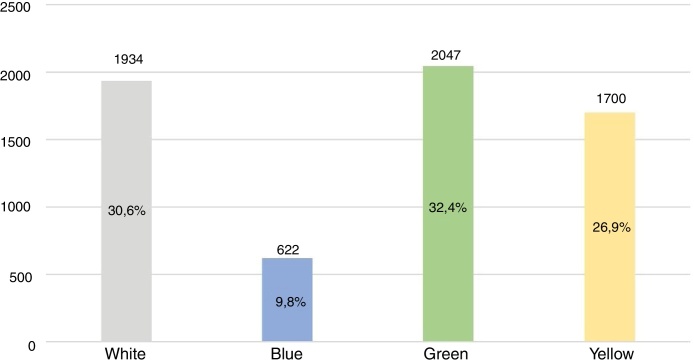
Classifications of teledermatology service reports in Florianopolis in 2016 and 2017*.

*Source*: Santa Catarina State Integrated Telemedicine and Telehealth System – Santa Catarina State Health Department. * Red classification was not represented in the graph because it presented only one record in 2016 and 2017.

Regarding the teledermatology service, an important element in the organization of referrals to the dermatology specialty, there was a predominance of classifications with need for secondary referral, *i.e*., green and yellow, representing 59.3% (Fig. 1).

In the biennium analyzed, the dermatology service presented the most prevalent diagnoses as “Other malignant neoplasms of skin” (C44), “Skin changes due to chronic exposure to nonionizing radiation” (L57), and “Psoriasis” (L40) (Table 3).

**Table 3:** Main categories from the International Statistical Classification of Diseases – 10th Revision (ICD-10) treated in the dermatology service of Florianópolis, in 2016 and 2017.

Rank	ICD-10		*n*	%
1	C44	Other malignant neoplasms of skin	2906	14.75
2	L57	Skin changes due to chronic exposure to nonionizing radiation	2010	10.20
3	L40	Psoriasis	1380	7.00
4	L82	Seborrheic keratosis	1072	5.44
5	L81	Other disorders of pigmentation	1020	5.17
6	D22	Melanocytic naevi	944	4.79
7	L70	Acne	882	4.47
8	L25	Unspecified contact dermatitis	454	2.30
9	B07	Viral warts	346	1.76
10	B35	Dermatophytoses	344	1.74
11	L80	Vitiligo	326	1.65
12	L63	Alopecia areata	226	1.15
13	L71	Rosacea	218	1.11
14	L72	Follicular cysts of skin and subcutaneous tissue	176	0.89
15	D18	Hemangioma and lymphangioma, any site	160	0.81
Total			19,694	100.00

With regard to ages of attended patients, the authors identified the prevalence of the ICD categories: atopic dermatitis (L20) in the age group of 0 to 12 years, with 16.7% of visits – table 4; acne (L70) in the 13–24 year-old group, with 31.9% – table 5; and other malignant neoplasms of the skin (C44), in the age groups of 25–59 years and 60 years or older (Table 6, Table 7).

**Table 4:** Main categories from the International Statistical Classification of Diseases – 10th Revision (ICD-10) in the age group of 0 to 12 years, treated in the dermatology service of Florianópolis, in 2016 and 2017.

0–12 years				
Rank	ICD-10		*n*	%
1	L20	Atopic dermatitis	52	16.7
2	D22	Melanocytic naevi	30	9.7
3	L98	Other disorders of skin and subcutaneous tissue	24	7.7
4	B08	Other viral infections characterized by skin and mucous membrane lesions	22	7.1
5	L30	Other dermatitis	22	7.1
6	L81	Other disorders of pigmentation	16	5.1
7	L70	Acne	14	4.5
8	D18	Hemangioma and lymphangioma, any site	12	3.8
9	B07	Viral warts	12	3.8
10	Q82	Other congenital malformations of skin	12	3.8
11	L25	Unspecified contact dermatitis	12	3.8
12	L40	Psoriasis	10	3.2
13	L80	Vitiligo	8	2.6
14	L11	Other acantholytic disorders	8	2.6
15	L72	Follicular cysts of skin and subcutaneous tissue	6	1.9
Total			310	100

**Table 5:** Main categories from the International Statistical Classification of Diseases – 10th Revision (ICD-10) in the age group of 13 to 24 years, treated in the dermatology service of Florianópolis, in 2016 and 2017.

13–24 years				
Rank	ICD-10		*n*	%
1	L70	Acne	620	31.9
2	L98	Other disorders of skin and subcutaneous tissue	280	14.4
3	L40	Psoriasis	130	6.6
4	D22	Melanocytic naevi	110	5.6
5	B07	Viral warts	74	3.8
6	L20	Atopic dermatitis	56	2.8
7	L25	Unspecified contact dermatitis	50	2.5
8	L81	Other disorders of pigmentation	50	2.5
9	L80	Vitiligo	44	2.2
10	L63	Alopecia areata	44	2.2
11	B36	Other specified superficial mycoses	42	2.1
12	L73	Other follicular disorders	38	1.9
13	L90	Atrophic disorders of skin	30	1.5
14	L91	Hypertrophic disorders of skin	28	1.4
15	L11	Other acantholytic disorders	24	1.2
Total			1942	100

**Table 6:** Main categories from the International Statistical Classification of Diseases – 10th Revision (ICD-10) in the age group of 25–59 years, treated in the dermatology service of Florianópolis, in 2016 and 2017.

25–59 years				
Rank	ICD-10		*n*	%
1	C44	Other malignant neoplasms of skin	1122	11.1
2	L40	Psoriasis	856	8.5
3	L81	Other disorders of pigmentation	704	7.0
4	D22	Melanocytic naevi	674	6.7
5	L57	Skin changes due to chronic exposure to nonionizing radiation	580	5.7
6	L82	Seborrheic keratosis	354	3.5
7	L25	Unspecified contact dermatitis	274	2.7
8	L70	Acne	270	2.7
9	B35	Dermatophytoses	230	2.2
10	L80	Vitiligo	218	2.1
11	B07	Viral warts	188	1.8
12	L63	Alopecia areata	172	1.7
13	L71	Rosacea	162	1.6
14	L93	Lupus erythematosus	118	1.1
15	L72	Follicular cysts of skin and subcutaneous tissue	114	1.1
Total			10,092	100

**Table 7:** Main categories from the International Statistical Classification of Diseases – 10th Revision (ICD-10) in the age group of 60 years and older, treated in the dermatology service of Florianópolis, in 2016 and 2017.

60 years and older				
Rank	ICD-10		*n*	%
1	C44	Other malignant neoplasms of skin	1784	24.2
2	L57	Skin changes due to chronic exposure to nonionizing radiation	1426	19.4
3	L98	Other disorders of skin and subcutaneous tissue	1270	17.2
4	L82	Seborrheic keratosis	718	9.7
5	L40	Psoriasis	384	5.2
6	L81	Other disorders of pigmentation	250	3.4
7	D22	Melanocytic naevi	130	1.7
8	L25	Unspecified contact dermatitis	118	1.6
9	D03	Melanoma in situ	98	1.3
10	L90	Atrophic disorders of skin	96	1.3
11	B35	Dermatophytoses	92	1.2
12	B07	Viral warts	72	1.0
13	L29	Pruritus	64	0.8
14	L80	Vitiligo	56	0.7
15	L71	Rosacea	56	0.7
Total			7350	100

Considering ICD-10 category groups, there are clear divergences among the PHC and dermatology profiles. In PHC, there was a higher prevalence of the groups other skin infections (25.65%); dermatitis and eczema (21.57%), and skin and subcutaneous tissue infections (14.23%). Whereas in dermatology, the most prevalent groups were other skin and subcutaneous tissue disorders (34.37%); neoplastic skin diseases (22.28%), and radiation-related disorders of the skin and subcutaneous tissue (10.39%; Table 8).

**Table 8:** Groups of International Statistical Classification of Diseases – 10th Revision (ICD-10) categories treated in the primary health care (PHC) and dermatology services, during 2016 and 2017 in Florianopolis.

Groups	PHC		Dermatology		RR	95% CI	*p*-Value^a^
	*n*	%	*n*	%			
Skin and subcutaneous tissue infections (L00–L08)	7866	14.23	42	0.21	3.50	2.28–5.38	<0.001
Bullous disorders (L10–L14)	161	0.29	102	0.52	2.49	1.75–3.53	<0.001
Dermatitis and eczema (L20–L30)	11,922	21.57	1192	6.05	1.00	0.91–1.08	<0.001
Papulosquamous disorders (L40–L45)	1662	3.01	1600	8.12	1.07	0.97–1.18	0.125
Urticaria and erythema (L50–L54)	1450	2.62	186	0.94	6.07	4.89–7.54	<0.001
Radiation-related disorders of the skin and subcutaneous tissue (L55–L59)	781	1.41	2046	10.39	0.14	0.13–0.16	<0.001
Disorders of skin appendages (L60–L75)	6892	12.47	2038	10.35	1.14	1.06–1.22	<0.001
Other skin and subcutaneous tissue disorders (L80–L99)	6249	11.31	6768	34.37	0.85	0.81–0.89	<0.001
Neoplastic skin diseases	3706	6.7	4388	22.28	0.71	0.67–0.75	<0.001
Other skin disorders	117	0.21	142	0.72	0.67	0.48–0.96	0.028
Leprosy	281	0.51	198	1.01	2.01	1.55–2.60	<0.001
Other skin infections	14,178	25.65	992	5.04	2.04	1.86–2.23	<0.001
Total	55,265	100.00	19,694	100.00	–	–	–

a Chi-squared test. RR, relative risk; 95% CI, 95% confidence interval.

## Discussion

Dermatological diseases represent more than 2000 pathological conditions in medicine, impacting quality of life and social relations worldwide. Despite this relevance, there is a tendency to not value these conditions in the national and global health scenario.^1^, ^2^, ^12^, ^17^ In Brazil, this neglect can be noted both in health policies and planning, as well as in scientific literature, which needs more studies about the epidemiological spectrum of these morbidities at different health care levels.

In doing so, this study evaluated nosological profile of skin diseases in PHC and dermatology services, in order to identify the prevalence of these conditions in these scenarios, thus understanding their influence on the requirements of medical practice. From the collected data, a distinct profile among the researched scenarios is notable, with a predominance of diagnoses related to infections, dermatitis, and eczema in PHC – generally acute and less complex cases – and diagnoses such as radiation-related disorders, neoplastic diseases, psoriasis, vitiligo and rosacea in dermatology – more complex and more specific conditions that require greater intrinsic knowledge of the field.

PHC represents the “gateway” and is an organizing element in health systems,^18^ receiving most of the dermatological disorders among its consultations. In light of this, in 2016 and 2017, the study shows that every 20 medical consultations in PHC had a dermatological disease as main diagnostic record, which represents about 5% of consultations performed. This result is similar to previous studies conducted in other countries, such as India (Poseidon Research),^19^ the United States (National Ambulatory Medical Care Survey: 2016),^20^ and Israel,^21^ but differs from Lowell et al.,^22^ who indicated one-third dermatological diagnoses in PHC consultations. Some patients show negligence regarding skin conditions, delaying medical assistance, which could be an explanation for the divergence among these studies. According to Penha et al.,^3^ their negligence may be related to lack of knowledge about skin diseases, fear, and/or shame of social stigmas attributed to these conditions.

Regarding the nosological profile in PHC, the most prevalent ICD groups were shown to be “Dermatitis and eczema” and “Skin and subcutaneous tissue infections,” with emphasis on the ICDs: “Scabies” (B86) and Impetigo (L01), which agrees with Lim et al. in 2017 and Lowell et al. in 2001, who demonstrated skin infections as one of the main skin pathology groups assisted in PHC.

In order to identify the epidemiological profile of dermatology services in Brazil, the Brazilian Society of Dermatology (BSD) conducted two large studies among its associated dermatologists, in 2006^17^ and 2018.^2^ These studies gathered clinical and demographic data about patients treated by dermatologists nationwide, showing some similarities and divergences in relation to the findings from Florianopolis.

In these studies, similarities are related to the high prevalence of diagnoses such as “Other disorders of pigmentation” (L81), “Skin changes due to chronic exposure to nonionizing radiation” (L57), “Non-melanoma cancer” (C44, C80), “Superficial mycoses” (B35-B36), and “Photoaging” (L57),^2^, ^17^ indicated by BSD findings, in agreement with the most prevalent diagnoses noted in Florianopolis, which were “Other malignant neoplasms of skin” (C44) and “Skin changes due to chronic exposure to nonionizing radiation” (L57).

Comparing the dermatology service of Florianopolis to other services of South Brazil, similar findings highlighting the main diagnoses “Non-melanoma cancer” (C44, C80), “Other disorders of pigmentation” (L81), and “Actinic keratosis” (L57.0) can be observed. This similarity reinforces the influence of regional sociodemographic and climatic contexts on the epidemiological profile of dermatological diseases in Brazil.^8^

It is emphasized that the prevalence of “Acne” in this research was lower when compared to BSD findings,^2^, ^17^ which could indicate a tendency to manage these conditions in PHC with teledermatology support. Another possible explanation could be the patients’ age profile, which showed a lower proportion of people in the age group of 13–24 years compared to other age groups; “Acne” has a high prevalence among young people that may influence the representativeness in data collected.

Concerning the 60 years or older group, it showed higher prevalence of “Other malignant neoplasms of skin” (C44) and “Skin changes due to chronic exposure to nonionizing radiation” (L57), reinforcing the influence of chronic radiation exposure in the triggering of skin diseases. From this perspective, the authors emphasize the relevance of attention to elderly patient's skin, in light of increased skin fragility and loss of barrier function predisposing to infections in this age group, as well as the fact that dermatological complaints are not usually the main reason to seek medical assistance.^23^

Regarding the teledermatology service, when analyzing the risk classification profile assigned by the service, it was noted that about 60% of risk classifications had referral criteria to dermatology, diverging from Tandjung et al.,^24^ who reported 36.8% of consultations with referral criteria. Thus, regarding divergences among PHC and dermatology secondary care nosological profiles, the authors emphasize the importance of integrating health care levels in order to promote the reorganization of referrals, as well as the early diagnosis of dermatological disorders with greater complexity and specificity, which require specialized clinical management.

As a side note, the results found come exclusively from public health services, which may represent an influencing factor in nosological profile – attenuating, for example, the cosmiatric component of dermatology. The BSD^2^ demonstrates significant differences in the demands of public and private dermatology services, with predominance of surgeries and cosmiatry in private consultations, and diseases such as non-melanoma skin cancer, leprosy, and psoriasis in public services. Moreover, it should be emphasized that when comparing these findings to previous studies, the ethnic, socioeconomic, and geoclimatic diversity among Brazilian regions may represent a confounding bias.

Concerning study limitations, the authors highlight the data loss due to “under-registration” caused by both deficiencies in the electronic medical records system and the tendency to underestimate dermatological complaints in health services. Another aspect to be considered is the poor specificity of some GPs’ records, adopting very broad and generic ICDs for their diagnoses, which could be associated with a certain level of diagnostic insecurity or by the institutional/professional culture itself.

It is also noteworthy that this study analyzed medical care records, *i.e*., some patients may have been seen several times by the same professional for different demands or the same demand in return visits. The variables adopted were limited by both the medical records system, which did not allow surveys of some data, and by the research focus. Further analysis may increase the number of variables evaluated, identifying dermatological diagnoses in different genders, skin phototypes, and social strata.

## Conclusion

This study noted different nosological profiles in medical care provided by general practitioners and dermatologists, in PHC and dermatology scenarios, respectively. Thus, the complementary and interdependent character among these professionals is emphasized, as well as the different attributions and skills required from them in each of these settings.

From this perspective, the role of the GP as PHC care coordinator and gatekeeper is highlighted, responsible for managing more prevalent and less complex conditions, referring to dermatology the more complex and rare cases, through the teledermatology service – an important organizing element of referral flow to dermatology secondary care. Therefore, dermatologists – with more technical and scientific experience in this field – should be regarded as focused specialists responsible for specialized clinical care in the health system.

## Financial support

None declared.

## Authors’ contributions

Iago Gonçalves Ferreira: Statistical analysis; approval of final version of the manuscript; conception and planning of the study; drafting and editing of the manuscript; collection, analysis, and interpretation of data; critical review of the literature; critical review of the manuscript.

Dannielle Fernandes Godoi: Approval of final version of the manuscript; conception and planning of the study; participation in design of the study; critical review of the manuscript.

Elaine Regina Perugini: Approval of final version of the manuscript; conception and planning of the study; drafting and editing of the manuscript; collection, analysis, and interpretation of data; participation in design of the study; critical review of the literature; critical review of the manuscript.

## Conflicts of interest

None declared.
